# Uniportal video-assisted thoracoscopic surgery following neoadjuvant chemotherapy for locally-advanced lung cancer

**DOI:** 10.1186/s13019-018-0714-9

**Published:** 2018-04-24

**Authors:** Zhiqiang Yang, Chunbo Zhai

**Affiliations:** Department of Thoracic Surgery, Weifang People’s Hospital, Shandong Province, Weifang, 261041 China

**Keywords:** Lung cancer, Neoadjuvant chemotherapy, Uniportal video-assisted thoracoscopic surgery (U-VATS)

## Abstract

**Background:**

Several retrospective studies have confirmed that video-assisted thoracoscopic surgery (VATS) following neoadjuvant chemotherapy is a safe and feasible treatment for advanced non-small cell lung cancer patients. As a minimally invasive technique, VATS usually leads to better clinical outcomes and better compliance with adjuvant treatment than conventional thoracotomy. Uniportal VATS (U-VATS) as an alternative option to conventional multi-port VATS has attracted much attention recently because reduced number and size of incisions may help to decrease inflammatory response and reduce postoperative pain for patients. However, rarely studies have reported the application of U-VATS following neoadjuvant chemotherapy for the treatment of advanced lung cancer patients.

**Methods:**

A total of 29 lung cancer patients undergoing VATS following neoadjuvant chemotherapy were included in this study. The clinical data of these patients were retrospectively analyzed, including the preoperative neoadjuvant chemotherapy plan, surgical effect, postoperative complications, operation time, operative blood loss, number of lymph nodes dissected and postoperative mortality.

**Results:**

All patients underwent VATS following two cycles of neoadjuvant chemotherapy. Among these patients, 26 completed U-VATS, two were converted to triple-port VATS, and one was converted to open thoracotomy. The operation time ranged from 120 min to 300 min (mean: 160 ± 38.5 min); the operative blood loss was 50–500 ml (mean:167.8 ± 78.4 ml); the number of lymph nodes dissected was 16–28 (mean: 21.9 ± 3.7); the postoperative drainage time was 3–13 d (mean: 5.6 ± 1.9 d); and the postoperative hospital stay was 6–16 d (7.7 ± 1.9 d). Postoperative complications occurred in five (17.2%) patients, including three cases of respiratory infection, one case of air leakage (more than two weeks), and one case of wound infection. In addition, the 30- and 90-day postoperative mortality was zero.

**Conclusion:**

U-VATS following neoadjuvant chemotherapy is feasible and safe for the treatment of advanced lung cancer patients.

## Background

Recent reports indicate that preoperative neoadjuvant chemotherapy or chemoradiotherapy can improve the completeness of resection and survival in patients with limited small-cell lung cancer (SCLC) and advanced non-small cell lung cancer (NSCLC) [1, 2]. However, tissue adhesions and increased vascular fragility after neoadjuvant chemotherapy could increase the risk of surgery, the incidence of postoperative complications and mortality [3].

Conventional open thoracotomy is associated with severe trauma and a lengthy recovery. Its tolerability in patients is very poor after neoadjuvant chemotherapy [4]. However, VATS lobectomy has the advantages of minimal trauma, less pain, quicker recovery and fewer complications [5]. Several retrospective studies have confirmed that VATS following neoadjuvant chemotherapy is a safe and feasible treatment for advanced NSCLC patients [6, 7]. Furthermore, uniportal VATS as an alternative option to conventional multi-port VATS has attracted much attention recently [8]. However, rarely studies have reported the application of U-VATS following neoadjuvant chemotherapy for the treatment of locally- advanced lung cancer patients [9]. The aim of the current study is to investigate the safety and feasibility of U-VATS following neoadjuvant chemotherapy for the treatment of advanced lung cancer patients.

## Methods

### Patient information

From June 2015 to October 2016, a total of 29 lung cancer patients (18 male and 11 female) with stage IIB-IIIB were included in this study. The mean age of these patients was 53.8 ± 6.4 years (range from 47 to 66 years). All patients were recruited from Weifang People’s Hospital in China. The detailed clinical and pathological features of these patients are shown in Table 1.

**Table 1:** Patient’s clinical and pathological features

Characteristic	No. (%)
Gender	
Male	18 (62.1)
Female	11 (37.9)
Histological types	
Adenocarcinoma	16 (55.2)
Squamous cell carcinoma	8 (27.6)
Small cell carcinoma	5 (17.2)
Clinical TNM staging	
IIB	8 (27.6)
IIIA	18 (62.1)
IIIB	3 (10.3)
Clinical TNM staging approach	
PET-CT	29 (100)
Definite diagnosis of histological types	
CT-guided biopsy	19 (65.5)
Bronchoscopy	10 (34.5)

All patients were diagnosed before preoperative chemotherapy. The histopathological diagnosis was confirmed by fibrobronchoscopy and percutaneous needle biopsy. The NSCLC types included adenocarcinoma (*n* = 16, 55.2%), squamous cell carcinoma (*n* = 8, 27.6%) and SCLC (*n* = 5, 17.2%). The clinical stages were assessed and confirmed through preoperative positron emission tomography–computed tomography (PET-CT). The preoperative examination also included bronchoscopy, brain MRI and cardiopulmonary function tests. Tumor, node and metastasis (TNM) staging was performed based on the 2009 UICC staging criteria (7th edition) [10], with eight cases of stage IIB (27.6%), 18 cases of stage IIIA (62.1%) and three cases of stage IIIB (10.3%).

### Neoadjuvant chemotherapy

The neoadjuvant chemotherapy treatment regimens of patients were selected based on the pathologic type. A total of sixteen adenocarcinoma patients received DP (docetaxel + cisplatin), eight squamous cell carcinoma patients received GP (gemcitabine + cisplatin), and five SCLC patients received EC (Etoposide + carboplatin, Table 2). All patients underwent two cycles of preoperative neoadjuvant chemotherapy. Four weeks after neoadjuvant chemotherapy treatment, the patients underwent another chest enhanced CT. If no lesion progression or reduction in staging was confirmed by CT, VATS was performed. The average time from the end of the neoadjuvant chemotherapy therapy to the operation was 35.5 ± 2.8 d.

**Table 2:** Neoadjuvant chemotherapy treatment regimens and evaluation of their effectiveness

Neoadjuvant treatment regimen	Effectiveness, n (%)		
	CR	PR	SD
GP	–	12	4
DP	–	5	3
EC	–	5	–

GP (gemcitabine + cisplatin): gemcitabine 1000 mg/m^2^, intravenous infusion, d1, d8; total cisplatin 90 mg/m^2^, intravenous infusion, d2–5, 21 days as one cycle; DP (docetaxel + cisplatin), docetaxel injection, intravenous infusion, 75 mg/m^2^, d1, total cisplatin 90 mg/m^2^, intravenous infusion, d2–5, 21 days as one cycle; EC (Etoposide,VP-16 + carboplatin), carboplatin intravenous infusion, d1, AUC (area under the curve) method to calculate the carboplatin dose, AUC = 5.5, total etoposide 100 mg/m^2^,d2–6, 21 days as one cycle

### VATS technique

The patients received general anesthesia in a contralateral supine position and underwent double-lumen endotracheal intubation. The surgeon and an assistant stood on the ventral side of the patient, and another assistant stood on the dorsal side of the patient. The operation hole was typically positioned between the middle axillary line and the anterior axillary line. In addition, an upper or lower lobectomy was usually performed via the 5th intercostal space, and a middle lobectomy was usually performed at the 4th intercostal space, with a 3–5-cm long incision. To enlarge the incision, a lap-protector was used rather than a rib spreader. The lung fissure and blood vessels were carefully separated referenced to their development on the upper lobe. In patients with an intractable left upper pulmonary artery, the pulmonary trunk was preferentially separated. In addition, the middle and lower lobectomies were performed in the following order: veins, lobar bronchia, arteries and lung fissure. Simultaneously, the lymph nodes in stations 10 and 11 were cleared away. After the lobectomy, a mediastinal lymph node dissection was systematically performed (stations 4, 5, 6, 7, 8, and 9 on the left; stations 2, 3, 4, 7, 8, and 9 on the right). Additionally, a 28F drainage tube was placed through the surgical incision after the operation.

### Statistical analysis

Statistical analyses were applied via SPSS software (version 17.0). Student’s t-test was performed to compare measurement data, whereas the χ2 test was utilized to compare enumeration data. A *P*-value of less than 0.05 was considered statistically significant (two sides).

## Results

### Clinical efficacy and toxic effects of neoadjuvant chemotherapy therapy

All 29 patients completed two cycles of preoperative neoadjuvant chemotherapy. Among the 29 patients who received neoadjuvant chemotherapy, 12 adenocarcinoma, 5 squamous cell carcinoma and 5 SCLC patients were radiologic partly response (PR), and another four adenocarcinoma and three squamous cell carcinoma patients exhibited a stable disease (SD) after neoadjuvant chemotherapy therapy (Table 2). Among the five SCLC patients, two exhibited a pathological complete response (CR, Table 2).

### Surgical results and complications

Among the 29 patients, 28 underwent successful VATS with systematic lymph node dissection. Among these patients, 26 underwent U-VATS, two were converted to triple-port VATS because of compact perivascular lymph nodes, and one was converted to open thoracotomy due to bleeding during the operation. The operation time ranged from 120 min to 300 min (mean: 160 ± 38.5 min); the operative blood loss was 50–500 ml (mean: 167.8 ± 78.4 ml); the number of the dissected lymph nodes was 16–28 (mean: 21.9 ± 3.7); the postoperative drainage time was 3–13 d (mean: 5.6 ± 1.9 d); and the postoperative hospital stay was 6–16 d (7.7 ± 1.9 d, Table 3). Postoperative complications occurred in five (17.2%) patients, including three cases of respiratory infection, one case of air leakage (more than two weeks), and one case of wound infection. In addition, both the 30- and 90-day postoperative mortality were zero.

**Table 3:** Surgical outcomes of patients undergoing U-VATS

Item	No. (%) or range
Lobe resection	
Right upper lobectomy	9 (31.1)
Right middle lobectomy	2 (6.9)
Right lower lobectomy	7 (24.1)
Left upper lobectomy	5 (17.2)
Left lower lobectomy	6 (20.7)
Postoperative TNM staging	
No tumor	2 (6.9)
IA	1 (3.5)
IB	3 (10.3)
IIA	7 (24.1)
IIB	10 (34.5)
IIIA	5 (17.2)
IIIB	1 (3.5)
Operation time (min)	160 ± 38.5
Intraoperative blood loss (ml)	167.8 ± 78.4
No. of lymph nodes dissected	21.9 ± 3.7
Postoperative chest tube duration (days)	5.6 ± 1.9
Postoperative hospital stay (days)	7.7 ± 1.9
Time to chemotherapy (days)	35.5 ± 2.8

## Discussion

In recent years, VATS lobectomy has been used worldwide and has been increasingly used to treat locally- advanced NSCLC [6, 11]. However, VATS following neoadjuvant chemotherapy is still a challenge. As reported, the incidence of postoperative complications after neoadjuvant chemotherapy or chemoradiotherapy is as high as 35–43.5% [3, 12]. In addition, the main cause of postoperative complications was tissue adhesions, indistinct interface and increased fragility of the blood vessels after neoadjuvant chemotherapy. Hence, safety after neoadjuvant chemotherapy is always the focus of surgeons. With the development of the thoracoscopic technique, the safety and feasibility of complete VATS (cVATS) following neoadjuvant chemotherapy have been increasingly reported [2, 6]. Huang et al [6] reported that the incidence of postoperative complications and perioperative mortality after cVATS was 9.5% and 2.4%, respectively, which was consistent with the outcomes of open thoracotomy.

U-VATS is a newly developed technology based on VATS. Since Rocco et al [13] first reported the use of lung wedge resection via U-VATS, the indications of U-VATS in diagnosis and treatment of lung disease have been extended. Very recently, Gonzalez-Rivas et al [14–16] used U-VATS to complete various complex and highly difficult procedures, including segmentectomy, bronchial sleeve lobectomy and double-sleeve (vascular and bronchial) lobectomy. Furthermore, Gonzalez-Rivas et al [9] and Fan et al [17] confirmed the safety and feasibility of U-VATS for advanced NSCLC patients. However, the use of U-VATS in treating advanced NSCLC following neoadjuvant chemotherapy, is still a challenge for most thoracic surgeons.

The mean operation time was 160 ± 38.5 min, the operative blood loss was 167.8 ± 78.4 ml and the number of lymph nodes dissected was 21.9 ± 3.7, which are all consistent with those reported by previously published studies [9, 17, 18]. However, the mean postoperative drainage time (5.6 ± 1.9 d) and postoperative hospital stay (7.7 ± 1.9 d) were longer than those reported by previously published studies [9, 17, 18]. The cause of the longer times may be related to the standard for removing the chest tube. In our current study, the chest tube was removed when the drainage was less than 100 ml/24 h. However, Zhang et al [19] compared the safety and feasibility of early chest tube removal (removal of chest tube when drainage is less than 300 ml/24 h) to the traditional management method (The chest tube was removed when the drainage is less than 100 ml/24 h), and they confirmed that early removal of the chest tube after lobectomy is feasible and safe. Additionally, the early removal of the chest tube could lead to a shorter hospital stay and reduce morbidity without increasing the risk of complications. Hence, we hypothesized that the traditional standard for chest tube removal may have contributed to the longer postoperative drainage time and hospital stay.

In our current study, the incidence of and mortality from postoperative complications were 17.2% and zero, respectively. Lung infection was typically the most common postoperative complication. Villamizar et al [20] reported that prior chemotherapy could predict increased complications based on a multivariable analysis, whereas Gonzalez-Rivas et al [9] and Fan et al [17] showed that VATS in advanced NSCLC patients after neoadjuvant chemotherapy was not associated with an increase in the incidence of postoperative complications. In addition, our results were similar as those reported by Gonzalez-Rivas et al [9] and Fan et al. [17] They also reported that reduced access trauma, a better view and the skills and experience of the surgeon may contribute to the low incidence of postoperative complications.

Among the 29 patients who underwent VATS following neoadjuvant chemotherapy, two (6.9%) patients were converted to triple-port VATS, and one (3.4%) patient was converted to open thoracotomy, which were consistent with previously studies [6, 9, 17]. Tissue adhesions, indistinct interface and increased fragility of the blood vessels after neoadjuvant chemotherapy were significant causes of operative blood loss. Additionally, mistakes during the operation could lead to blood loss as well. For example, injury of the vascular wall from violent dissection of the blood vessels or a forced pass of the stapler may cause avulsion of the vascular roots when the length of the dissected blood vessels was insufficient. When rupture of the veins and bronchus occurs, violent tension from the assistants could also cause the rupture of blood vessels.

Another cause of conversion to thoracotomy was perivascular and peribronchial lymph node calcification. As Park et al [21] reported in a retrospectively study, 41% of conversions may be due to the anthracofibrosis of hilar lymph nodes or hilar adhesions. They also analyzed the CT scans of patients and found that 71% of patients suffered from different levels of hilar lymph node calcification. In our current study, two patients were not converted to open thoracotomy for the compact perivascular lymph nodes, but the operation could not be completed by U-VATS; two additional ports were added to finish the lobectomy. In addition, Wang et al [22] gained proximal and distal control of the artery before cutting the arteries and the application of a stapler at the end was an important way to ensure the safety and reliability of U-VATS in treating calcified lymph nodes.

To reduce the occurrence of operative blood loss, the following strategies were utilized: (I) Treat the part with the lowest risk of bleeding first during surgery, and avoid adherence to a certain order; (II) Dissect blood vessels extensively and expand the vascular space. Additionally, a silk thread could be used to hold the vessels when stapler was difficult to use; (III) When the risk of bleeding is high, freeing the root of the pulmonary artery in advance and temporarily blocking the artery could not only reduce the occurrence of operative blood loss but also reduce the time required to repair the ruptured blood vessels; (IV) For small blood vessels, cut after silk thread ligation was safer and more convenient; and (V) When there is gap fuzziness and dense adhesion between the blood vessels and bronchia such that blunt separation is difficult, we can temporarily cut the bronchia and suture the bronchial stump or use a stapler at the end after lung lobectomy.

Some limitations were unavoidable in this study. First, this was a retrospective study and thus it lacked prospective randomized controls. Second, the clinical stages of patients were assessed and confirmed through preoperative PET-CT without verification by endobronchial ultrasound or mediastinoscopy, which might generate biases on the accuracy of the clinical stages. As reported, PET-CT has high sensitivity, specificity and diagnostic accuracy in the mediastinal staging of patients, but PET-CT alone cannot judge the extent of mediastinal lymph node involvement [23]. Hence, the combination of PET-CT and other examination methods could improve the diagnostic accuracy of clinical stages. Third, due to the limited number of patient samples in this study, our conclusions in the manuscript need to be further validated with more patient samples in the future.

## Conclusions

In conclusion, our current study further confirmed the safety and feasibility of U-VATS following neoadjuvant chemotherapy for the treatment of advanced lung cancer patients considering the incidence of and mortality from operative and postoperative complications. Extensive experience, a solid foundation for VATS and rational operation procedures guarantee the successful completion of U-VATS. However, whether U-VATS could be more widely used and whether U-VATS is more advantageous than multiportal VATS or open thoracotomy still require further study.

## Abbreviations

CR: complete response
cVATS: complete VATS
DP: docetaxel + cisplatin
EC: Etoposide + carboplatin
GP: gemcitabine + cisplatin
NSCLC: non-small cell lung cancer
PET-CT: positron emission tomography–computed tomography
PR: partly response
SCLC: small-cell lung cancer
SD: stable disease
TNM: Tumor, node and metastasis
U-VATS: Uniportal VATS
VATS: video-assisted thoracoscopic surgery
